# Removal of Eroded Gastric Bands Using a Transgastric SILS Device

**DOI:** 10.1155/2013/852747

**Published:** 2013-12-16

**Authors:** C. Spitali, K. De Vogelaere, G. Delvaux

**Affiliations:** UZ Brussel, Brussels 1090, Belgium

## Abstract

*Background*. Laparoscopic adjustable gastric banding (LAGB) is a popular method for the treatment of morbid obesity. One of the most feared complications is gastric band erosion which occurs with a reported incidence of 0.3 to 14%. Intragastric migrated bands are best managed by endoscopic removal. Recent case studies reported successful endoscopic removal of intragastric migrated bands, but it is not always possible. We report our first experience with a transgastric removal of eroded bands using a Single Incision Laparoscopic Surgery (SILS) device. *Methods*. A patient who underwent gastric banding in the past (2007) presented with symptoms of epigastric pain and weight gain. Preoperative gastroscopy revealed stomach wall erosion with the gastric band partially (2/3) migrated into the gastric lumen. Attempts to remove the band by endoscopy were not successful. A laparoscopy was performed and multiple adhesions with evidence of inflammation was seen in the upper abdomen around the band. A SILS port was inserted through a 2 cm incision in the left hypochondrium with the internal ring of the port placed into the stomach through a small anterior gastrotomy. The band was cut in the stomach and removed. The anterior gastrotomy was closed. We had a perfect intragastric view of the gastric banding. *Results*. There were no intra- or postoperative complications. The patient was discharged on the fifth postoperative day on a gastric adapted diet. *Conclusion*. Removal of a gastric band after gastric erosion by SILS is feasible, safe, and effective. This is the first reported case of transgastric removal of eroded bands using an SILS device.

## 1. Introduction

Laparoscopic adjustable gastric banding (LAGB) is an effective treatment for morbid obesity.

One of the most serious complications associated with LAGB is intragastric band migration or band erosion through the stomach wall.

The best management of band erosion is unclear in the current published literature.

We present a case of gastric band erosion 4 years after placement.

We removed the band using an SILS device placed transgastrically.

## 2. Case Report

A 31-year-old female underwent laparoscopic gastric banding for morbid obesity 4 years ago.

A Lap band (Bio Entererics) was placed laparoscopically utilizing the pars flacida technique. Preoperatively there were no problems. The patient recovered well and was discharged one day after the intervention.

One year following the procedure, the patient presented to the emergency room with abdominal pain.

A abdominal CT scan was negative for leak or free air, but showed an inflammation around the port.

The band was deflated and the patient was admitted for observation. The patient recovered well and was discharged after 4 days.

The band was adjusted with saline several times, without any problem.

Four years following the procedure, the patient presented to our consultation with symptoms of epigastric pain and weight gain.

A UGI contrast study showed a slippage of the band.

**Figure 1:**
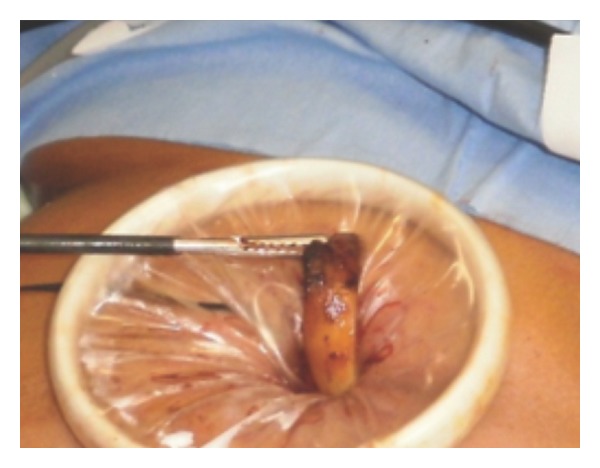
Endoscopy revealed stomach wall erosion with the gastric band partially (2/3) migrated into the gastric lumen.

Endoscopy revealed stomach wall erosion with the gastric band partially (2/3) migrated into the gastric lumen (Figure 1).

We decided to remove the eroded band.

The procedure was performed in the operating room with the patient under general anesthesia with endotracheal intubation.

Peroperative a gastroscopy was performed. Removal of the band by endoscopy was impossible because the band could not be cut with the material available for endoscopy.

A laparoscopy was performed and multiple adhesions with evidence of inflammation were seen in the upper abdomen around the band. There was no visualisation of the band.

**Figure 2:**
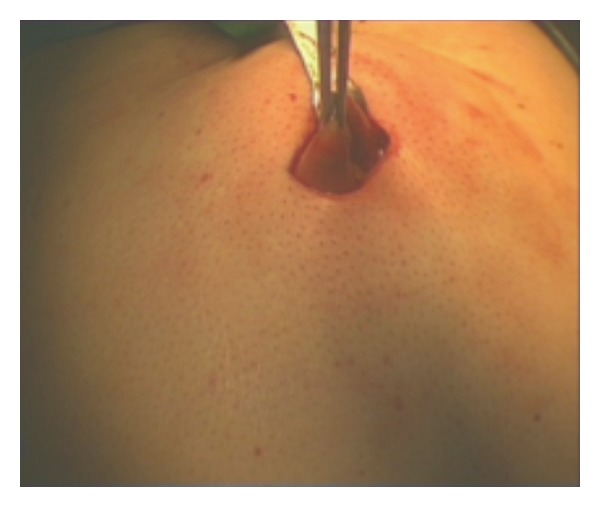
Stomach was grasped at the anterior site and brought outside the abdomen.

A 2 cm skin incision was made at the left hypochondrium. The stomach was grasped at the anterior site with straight forward instruments and brought outside the abdomen (Figure 2). The stomach was opened at the anterior site and the edge of the stomach was sutured at the edge of the skin with interrupted stiches. The internal ring of the SILS port (Allergan) was placed into the stomach, while the external ring was outside. Insufflation was started at 15 mmHg. We had a perfect view of the internal site of the stomach.

A standard 10 mm laparoscope was used for visualization and two 5 mm straight forward working instruments were inserted through the SILS port.

**Figure 3:**
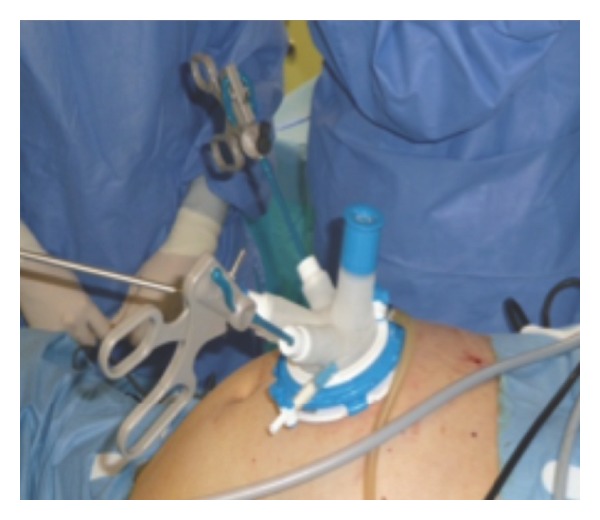
The band was cut in the stomach with scissors and removed.

The band was cut in the stomach with scissors and removed (Figure 3).

The SILS port was removed and the supporting stiches of the stomach were removed. The anterior gastrotomy was closed outside with a running suture of PDS 3.0 (Ehicon). The stomach was pulled back into the abdomen.

Methylene blue and air tests were performed during the operation to rule out leaks, and they were negative.

A Blake drain 19Fr (Ethicon) was placed near the gastrotomy.

A nasogastric tube was also left in place postoperatively.

The operation time was 60 minutes. There was no blood loss.

There were no intra- or postoperative complications.

The nasogastric tube and drain were removed on postoperative day 2 and the patient resumed oral dieting beginning by liquids.

The patient was discharged from the hospital on postoperative day 5.

At the postoperative control no complications were seen after 1 month.

## 3. Discussion

Laparoscopic adjustable gastric banding (LAGB) is an effective treatment for morbid obesity.

The complications reported for LAGB are infections of the port site, band slippage, pouch dilatation, and intragastric band migration or band erosion.

Gastric band erosion has a highly reported incidence of 0.3 to 14% [1].

Erosion usually presents as a late complication (1–3 years after intervention) but some series report erosion within the first weeks.

Early erosion is generally a technical problem due to unrecognized gastric perforation during surgery or an early infection.

Other theories for erosion as a late complication are gastric wall ischemia secondary to a tight band, peptic ulcer perforation, implantation of a contaminated device, binge eating, and purging [2].

A large spectrum of clinical presentations is possible. From loss of restrictive effect, unexplained weight gain to life threatening sepsis and multiorgan failure.

Diagnosis can be confirmed by upper gastrointestinal endoscopy. CT findings can be suggestive, if there is extraluminal air or periprosthetic infection [1].

Intragastric migrated bands are best managed by endoscopic removal. Recent case studies reported successful endoscopic removal of intragastric migrated bands, but it is not always possible [3]. The timing of removal is still unclear.

It is also recommended to wait at least 3 months before reinsertion of another band [1, 3].

Removal of a gastric band after erosion by an SILS port introduced in the stomach has never been reported before.

We report the first case of the use of transgastric SILS port to remove eroded gastric bands.

Removal of a gastric band after gastric erosion is feasible, safe, and effective.
